# “Fitspiration” on Social Media: A Content Analysis of Gendered Images

**DOI:** 10.2196/jmir.6368

**Published:** 2017-03-29

**Authors:** Elise Rose Carrotte, Ivanka Prichard, Megan Su Cheng Lim

**Affiliations:** ^1^ Burnet Institute Melbourne Australia; ^2^ School of Health Sciences Flinders University Adelaide Australia; ^3^ School of Public Health and Preventive Medicine Monash University Melbourne Australia; ^4^ Melbourne School of Population and Global Health The University of Melbourne Melbourne Australia

**Keywords:** social media, physical fitness, women’s health, men’s health, body image

## Abstract

**Background:**

“Fitspiration” (also known as “fitspo”) aims to inspire individuals to exercise and be healthy, but emerging research indicates exposure can negatively impact female body image. Fitspiration is frequently accessed on social media; however, it is currently unclear the degree to which messages about body image and exercise differ by gender of the subject.

**Objective:**

The aim of our study was to conduct a content analysis to identify the characteristics of fitspiration content posted across social media and whether this differs according to subject gender.

**Methods:**

Content tagged with #fitspo across Instagram, Facebook, Twitter, and Tumblr was extracted over a composite 30-minute period. All posts were analyzed by 2 independent coders according to a codebook.

**Results:**

Of the 415/476 (87.2%) relevant posts extracted, most posts were on Instagram (360/415, 86.8%). Most posts (308/415, 74.2%) related thematically to exercise, and 81/415 (19.6%) related thematically to food. In total, 151 (36.4%) posts depicted only female subjects and 114/415 (27.5%) depicted only male subjects. Female subjects were typically thin but toned; male subjects were often muscular or hypermuscular. Within the images, female subjects were significantly more likely to be aged under 25 years (*P*<.001) than the male subjects, to have their full body visible (*P*=.001), and to have their buttocks emphasized (*P*<.001). Male subjects were more likely to have their face visible in the post (*P*=.005) than the female subjects. Female subjects were more likely to be sexualized than the male subjects (*P*=.002).

**Conclusions:**

Female #fitspo subjects typically adhered to the thin or athletic ideal, and male subjects typically adhered to the muscular ideal. Future research and interventional efforts should consider the potential objectifying messages in fitspiration, as it relates to both female and male body image.

## Introduction

### Overview of Fitspiration

An estimated 79% of young people use some form of social media daily [1]. Research suggests that young people are increasingly turning to social media for information about health and health behavior norms [2]. In recent years, a new fitness trend has emerged, providing Web-based and social media–based content designed to inspire individuals to exercise and be healthy. “Fitspiration,” commonly shortened to “fitspo,” is the broad term used to describe this “fitness inspiration” [3]. Fitspiration on social media allows users to view exercise-related images and videos and communicate with like-minded individuals. It often contains exercise tips, recipes, and photographs of food or people (including professional photographs, self-portraits (“selfies”) and “before and after” images to highlight changes in weight or muscle). It is conceptually different from “thinspiration” (#thinspo), a form of media that deliberately promotes weight loss and thinness, and glorifies aspects of disordered eating behavior [4]. Social media users may follow dedicated fitspiration pages and profiles so that related content appears in their newsfeeds. Fitspiration-related social media posts are often tagged using hashtags—short words or phrases preceded by the hash or number symbol (#)—such as “#fitspo,” allowing social media users to easily search for posts related to this topic. A recent cross-sectional survey estimated that 31% of young Australians like or follow fitspiration on social media, with young women more than twice as likely as young men to like these pages [5].

### Impact of Fitspiration

Fitspiration is perceived to “model” ideas about health and fitness, shaping health beliefs and encouraging a “moral obligation” to achieve a particular body type among young women [6]. Experimental research has demonstrated negative effects from acute exposure to fitspiration among women, including increased negative mood and body dissatisfaction [7]. Acute exposure to fitspiration-style athletic ideal images (which depict a thin yet toned or muscular female body [8]) and exposure to “thinspiration”-style thin ideal images [9] predict body dissatisfaction and compulsive exercising among women at similar rates [10-12].

Meanwhile, contemporary men also commonly experience body dissatisfaction, particularly muscle dissatisfaction [13]. Exposure to traditional media (eg, print or magazine) muscular ideal images is associated with increased drive for muscularity and depression [14] and lowered muscle satisfaction among men [15]. Furthermore, exposure to images of male models actively engaging in sport has been shown to decrease men’s satisfaction with their fitness levels and overall appearance [16]. However, the impact of male fitspiration images on men remains unknown.

### Objectification Theory

Despite the focus on fitness, fitspiration images are argued to focus heavily on the appearance of the body and emphasize looks rather than body functionality [7]. According to objectification theory [17], this treatment of the body as an object (“objectification”) is common in Western society, occurs in many forms, and disproportionately affects women. A common form of objectification is sexual objectification, where the body is treated and visually inspected as a collection of sexually appealing body parts [17]. Past research has shown that over 50% of the time, traditional media presents the female form as a “sex object,” using a woman’s sexuality to sell a product via facial expression, the amount of skin shown, and sexually suggestive camera angles. This figure rises to approximately 76% when examining images of women in men’s magazines [18]. Furthermore, objectification may also be evident through facial prominence in images. Research suggests that images of men tend to focus on the head and face, whereas for women, the emphasis is on the whole body; this is known as “face-ism” [19]. A comprehensive analysis of objectification in fitspiration images would identify gender differences in these areas and provide avenues for future research.

### Fitspiration Content Analyses

Content analyses have important implications for policy, as they can identify which types of content are potentially beneficial or harmful, and the degree to which fitspiration may be targeting different demographics. Two recent studies analyzed fitspiration websites; finding that these websites contain similar content to thinspiration websites, mostly depict women (>90% of images), and include objectified and sexualized women who are objectively thin [3,20]. However, it is unclear whether any social media–based fitspiration was included in these website analyses. In addition, gendered comparisons were absent from these studies [3,20], presumably as the majority of website-based content depicted women. Due to the popularity of fitspiration on social media, and its potential to facilitate peer-based body comparisons and reinforce social norms around health and fitness [5], it is also important to also study social media–based fitspiration. Recently, Tiggemann and Zaccardo [21] examined fitspiration on the image-based social media platform Instagram, finding that although most of these images (67%) depicted women, around 29% of images depicted men. Using categorical measurements, they found that most female subjects were thin and visibly muscular, whereas most male subjects were of medium build with a high level of muscularity. A quarter of subjects were engaged in some exercise activity and the majority of both men and women were objectified.

Although these previous content analyses have provided a broad overview of the common appearance-based messages of fitspiration, they do not provide any inferential statistics related to potential differences in male and female images. Tiggemann and Zaccardo [21] provided limited description of body type, activity engagement, and objectification between male and female subjects; however, this was only on one media platform (Instagram). It is currently unclear whether social media fitspiration content is most commonly posted to Instagram or to another social media platform, and whether these patterns exist across different social media platforms such as Facebook, Twitter, and Tumblr. Instagram and Tumblr are highly visual platforms compared with Twitter and Facebook, which have more of a mix of text- and image-based content. Furthermore, Instagram and Tumblr have more female users than male users [22].

Although women are more likely than men to access social media–based fitspiration [5], since over a quarter of Instagram content appears to be aimed at men [21], it is also important to examine messages aimed at men. Prior studies of promuscularity websites indicate that these websites contain messages about rigid exercise and dietary practices and aspirations toward an “ideal” muscular body [23]. Such messages may contribute toward rates of body dissatisfaction, disordered eating, and compulsive exercise behaviors in men. Studying these gendered differences in social media fitspiration content by using inferential statistics to clearly identify significant differences, could help identify patterns of fitspiration messages, inform future experimental research, and help to develop and refine interventions aimed at men and women. Furthermore, no prior content analyses have explored the age of fitspiration subjects. It is important to determine whether fitspiration is targeting particular age groups. Of note, body image concerns tend to commence earlier in girls than boys [24], and younger people disproportionately access fitspiration [4].

### This Study

This study aimed to describe and identify the characteristics of fitspiration content posted across social media (ie, Instagram, Facebook, Twitter, and Tumblr) via a public hashtag with regards to body image messages, food or dieting messages, and exercise messages by gender of subjects (see Multimedia Appendix 1 for variable description). In accordance with objectification theory and media trends, it was hypothesized that (1) posts would more frequently appear on Instagram and Tumblr than Twitter and Facebook; (2) more posts would depict images of women than images of men; (3) women would be thinner whereas men would be more muscular; (4) posts depicting women would be more likely to demonstrate objectification through sexualized imagery, emphasis placed on the look of the full body, emphasis placed on specific body parts (stomach or buttocks), and fewer depictions of the face, and (5) women would appear younger than men.

## Methods

### Selection of Content

This content analysis involved analyzing recent posts with the “#fitspo” hashtag across 4 social media platforms: Instagram, Facebook, Twitter, and Tumblr. At 3 randomly generated timeslots, #fitspo was searched across Instagram, Tumblr, Facebook, and Twitter and all posts were extracted using screenshot methods over the next 10 minutes. No best practice tools are available for systematically searching social media, and various websites’ default search algorithms do not allow systematic searching. At the time of data collection, Facebook and Pinterest did not allow accurate searching of “most recent” tagged posts, instead showing a combination of recent and “popular” posts when searching a tag. Instagram allows searching of recent posts for public, but not private profiles, but does not allow searching of popular posts. To address these issues, the website tagboard.com was used, which tracks recent, public posts with hashtags across Instagram, Facebook, and Twitter. Tumblr allows searching of both recent and popular posts; all profiles are public, so this platform was searched directly. The authors were unable to search for recent posts on Pinterest, another platform of interest, so this platform was not included in the analysis.

### Coding Strategy

**Figure 1:**
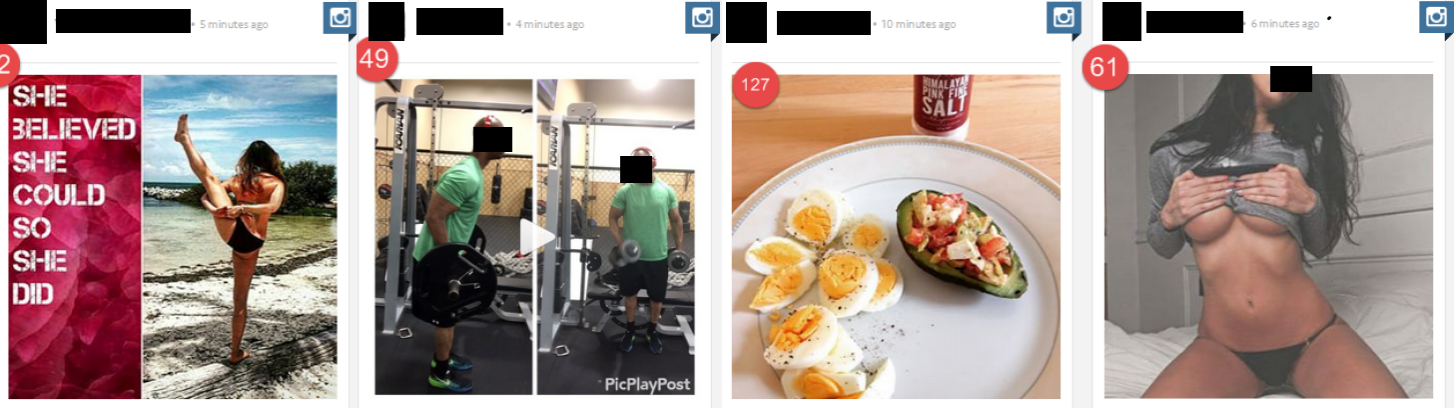
Examples of posts included in the content analysis. These posts demonstrate the following variables, among others: (L-R) full body, active exerciser, healthy eating, and sexualisation.

Given the emergent nature of research into this field, posts were analyzed with a codebook developed specifically for this project by the authors (see Figure 1 for example images and Multimedia Appendix 1 for codebook). This codebook was expanded from Boepple and Thompson’s [3], and Tiggemann and Zaccardo’s [21] analyses. Variables were also informed by objectification theory [17]. Twenty-eight variables were chosen by the authors. These included variables related to the relevance and structure of the post (eg, social media platform type, presence of a caption, presence of a photo or video), the theme of the post (exercise or food), number of people present and their apparent gender and age (in categories), whether the post depicted the subject’s face or body, whether the post emphasized the subject’s stomach or buttocks, and whether the post encouraged healthy eating or included a person actively exercising. For variables relating to the subject of the post, if multiple people of the same gender were present, coders were instructed to code variables related to the main subject of the post. This was determined by the structure of the post highlighting 1 subject (eg, 1 subject in the foreground). If this was unclear, the coder analyzed the subject on the left of the image (if viewing the image from the left to the right, this would be the first person viewed). For ease of interpretation, age was later collapsed into a binary variable of <25 or 25+ years for the gendered analysis.

Thinness and muscularity of the people in the images were rated using line drawing figure scales: the Figure Rating Scale (also known as Stunkard scale) for male and female thinness [25] and modified versions of this scale for male and female muscularity [26,27]. These scales use simple drawings of male and female figures to assess thinness and muscularity of men and women on scales of 1 (very thin or very little visible muscle) to 9 (very overweight or very muscular). Body parts emphasized were determined by visual cues such as proximity to the camera, cropping, and captions which drew attention to particular body parts.

Each post was analyzed by 2 independent coders. Both coders were trained in using the codebook before the analysis process using example posts not included in the analysis. Coders were instructed to assess the main messages or clear implications of the post, and to use any visible captions, hashtags, and comments to give context to the post. Coders were instructed to view videos, if possible, using links embedded in screenshots taken with tagboard.com. If this was not possible, coders rated the single frame video preview. Reliability was analyzed between the 2 coders using Cohen kappa for categorical variables (see Multimedia Appendix 1). Categorical variables included in the analysis exceeded the recommendation for a minimum value of κ=.60 [28] and percentage agreements for these variables ranged from 87.8% (for “Full Body”) to 99.5% (for “Platform”). Five categorical variables were removed due to low reliability. Reliability was analyzed for ordinal and interval-level variables using Spearman rho; these values were highly correlated, ranging from with ρ=.74 for thinness to ρ=.96 for number of women present. A third reviewer, the lead author, independently analyzed any discrepancies in accordance to the codebook with consideration of the 2 primary coders’ data, making the final decision and forming the final dataset.

### Gender Analysis

In posts containing men only and those containing women only, gender differences in mean thinness and muscularity ratings were analyzed using independent *t* tests, and chi-square or Fisher exact tests were used for analyzing categorical variables by gender of subjects. Cohen *d* or the phi coefficient was used to calculate effect sizes. Analyses were conducted in Stata version 13 (StataCorp LLC).

## Results

### Description of Content

Across the 30-minute composite study period, 476 social media posts tagged with “#fitspo” were retrieved across the 4 platforms. After removal of 61 irrelevant posts (κ=.73; see Multimedia Appendix 1), 415 of 476 posts (87.2%) were coded (Table 1). The most content was posted to Instagram (360/415, 86.8%), followed by Tumblr (39/415, 9.4%), Facebook (12/415, 2.9%), and Twitter (4/415, 1.0%) (κ=.98). Due to the relatively small number of posts across Tumblr, Facebook, and Twitter, variables were not compared by platform.

Most #fitspo posts contained a photograph or another image, and around 1 in 10 posts contained a video. More women appeared in posts than men, but men still appeared in nearly one third of posts; 40.5% (168/415) depicted at least one woman and 31.6% (131/415) depicted at least one man. In general, subjects of posts appeared to be young adults. Most posts were thematically related to exercise or fitness, although around 1 in 6 was thematically related to food (Table 1). Most messages about food (64/68, 94%) were found in posts without any people present, whereas most messages about exercise (245/295, 83.1%) were found in posts with at least one person present.

**Table 1:** Description of social media posts tagged with #fitspo (N=415).

Type of variable	Category	Level	Posts n (%)
Structure	Text	Motivational or inspirational quote or text	67 (16.1)
	Caption	Caption present	330 (79.5)
	Photo	Photo present	332 (80.0)
	Video	Video present	41 (9.9)
	Selfie	Post contains a selfie	112 (27.0)
Subjects	Person^a^	At least one person present	283 (68.2)
	Age (years)	<18	7 (1.7)
		18-24	104 (25.1)
		25-34	81 (19.5)
		35-44	12 (2.9)
		45+	1 (0.2)
		Multiple people of different ages	10 (2.4)
		Unclear	68 (16.4)
		Not applicable; no people present	132 (31.8)
	Gender^b^	Women only	151 (36.4)
		Men only	114 (27.5)
		Women and men	17 (4.1)
		Unclear	1 (0.2)
		Not applicable; no people present	132 (31.8)
Theme	Exercise	Thematically relates to exercise or fitness only	295 (70.1)
	Food	Thematically relates to food or eating only	68 (16.4)
	Both	Thematically relates to both food or exercise	13 (3.2)
	Neither	Thematically relates to neither food nor exercise	39 (9.4)

^a^Mean number of people present per post: 1.4 (SD 1.7, when at least one person present).

^b^Mean number of men present: 1.1 (SD 0.7); mean number of women present: 1.4 (SD 2.0, when at least one man or one woman were present, respectively).

### Gendered Analysis

Gendered analyses of posts depicting only men and posts depicting only women (265/415, 63.9%) were conducted. On the Figure Rating Scales, female subjects had significantly lower mean thinness scores (Mean 3.0, SD 1.2) than male subjects (Mean 4.4, SD 1.1), *P*<.001, Cohen *d*=−1.11, large effect. Female subjects also had significantly lower muscularity scores (Mean 4.1, SD 1.7) than male subjects (Mean 6.0, SD 1.2), *P*<.001, Cohen *d*=−1.30, large effect. Of note, these thinness and muscularity analyses were conducted only among posts where reviewers were able to assess thinness (n=126 for female subjects, n=89 for male subjects) and muscularity (n=116 for female subjects, n=94 for male subjects).

Categorical variables are presented in Table 2 (variable descriptions available in Multimedia Appendix 1). Posts containing women only were significantly more likely to display the subject’s full body than posts containing men only. Posts containing men only were significantly more likely to have their subject's face visible than posts containing women only. Nearly half of posts emphasized the subject’s stomach or contained an active exerciser, regardless of gender. Women were sexualized significantly more than men, although sexualization was common for both men and women. Posts containing women only emphasized the subject’s buttocks significantly more frequently than posts containing men only. Before and after images, and messages about food and healthy eating, were relatively rare regardless of gender. Messages about food and healthy eating were generally depicted in posts without any people present.

**Table 2:** Content of social media #fitspo posts by gender of subject when only one gender was present in the post.

Type of variable	Category	Female subjects only (n=151) n (%)	Male subjects only (n=114) n (%)	*P* value	Phi (φ)	Effect size
Age of subjects	<25 years	78 (70.9)	31 (37.8)	<.001	.33	Medium
**Structure of post**						
	Motivational text	11 (7.3)	11 (9.7)	.49		
	Caption	118 (78.2)	86 (75.4)	.60		
	Photo	135 (89.4)	98 (86.0)	.40		
	Video	18 (11.9)	21 (18.4)	.14		
	Selfie	66 (43.7)	38 (33.3)	.09		
**Theme of post**						
	Food only^a^	1 (0.7)	2 (1.8)	.58		
	Exercise only	126 (83.4)	104 (91.2)	.06		
**Objectification**						
	Face visible	76 (50.3)	77 (67.5)	.005	.17	Small
	Full body or nearly full body visible	98 (64.9)	51 (44.7)	.001	.20	Small
	Emphasis on stomach	69 (45.7)	47 (41.2)	.47		
	Emphasis on buttocks^a^	38 (25.2)	2 (1.8)	<.001	.71	Large
	Sexualization	72 (47.7)	33 (29.0)	.002	.19	Small
**Other variables**						
	Before or after^a^	3 (2.0)	1 (0.9)	.64		
	Healthy eating^a^	4 (2.7)	5 (4.4)	.51		
	Active exerciser	80 (53.0)	61 (53.5)	.93		

^a^Chi-square tests only performed with minimum frequency >5 per cell. Fisher exact test was used if cell frequency was 5 or fewer.

## Discussion

### Principal Findings

This study aimed to analyze the content of posts with the #fitspo hashtag over a composite 30-minute period across the social media platforms Instagram, Facebook, Twitter, and Tumblr, comparing messages of fitspiration by gender of the subject. As hypothesized, the vast majority of posts in this time period were posted to Instagram, followed by Tumblr, probably due to their highly visual nature. Similar to the findings of Tiggemann and Zaccardo [21], female subjects were more frequently depicted in fitspiration than male subjects; however, a third of posts depicted at least one male subject. This indicates that, in contrast to the female focus of previous fitspiration research (eg, [3,7]), it is likely that fitspiration is reaching men and may influence the body image, exercise, and health behaviors of male followers as well as female followers. As such, future research should investigate the potential impact of these images on men.

Posts depicting women, which are likely aimed at female social media users, typically depicted young adult women meeting either the thin ideal or the athletic ideal. Posts depicting very muscular women were also common, and may indicate an increase in the popularity of body building among women. Female subjects were frequently objectified and sexualized, with emphasis on the idealized look of their full body and body parts such as the stomach and buttocks. Considering the popularity of fitspiration among young women [5], and the detrimental effects on female body image observed previously [7,10], it is fair to assume that young women viewing this content are frequently exposed to images of thin and athletic ideal bodies, often sexualized, and that this content has the power to influence their body image and encourage exercise to alter their appearance. Furthermore, only half of the images of women actually depicted women’s faces (face-ism [19]), following a similar trend to that observed by Tiggemann and Zaccardo [21]. This trend may also be related to greater self-objectification, particularly in the context of social media, where the profile pictures of male users are more likely to depict their faces compared with female social media users [29].

Interestingly, men were frequently objectified, albeit in different ways to women. Although men had their faces in images more often than women, posts depicting men (presumably aimed at male social media users) depicted subjects that were highly muscular—significantly more muscular than female subjects. Images of men focused on stomachs at a similar frequency to women, but unlike women, men’s stomachs nearly always had visible abdominal muscles. Although not included in this analysis, images of men often emphasized visible biceps and pectoral muscles. Compared with posts with female subjects, in the images of men there was little emphasis on thinness or weight loss. This is aligned with current body ideals for men [13] and a general trend seen in the media of increased muscle mass in images of male bodies over time [30]. However, previous research indicates that young men access fitspiration on social media less frequently than young women [5]. There may be a small group of social media users posting content to the #fitspo hashtag who are very active and have a small but dedicated male audience. It is also possible that male social media users are accessing similar content but not on social media or do not consider this content to be fitspiration, and use different hashtags to label the fitness material they post (eg, #swoll which refers to swollen muscles).

Despite these concerns, it is possible that social media users viewing fitspiration are inspired to exercise, and that they view fitspiration in a positive manner. Qualitative research indicates that young female fitspiration users appreciate the ease of access to health and fitness information provided by fitspiration and generally choose to follow normalized and dominant health discourses [6]. Many posts were identified with balanced approaches to health and fitness. However, only half of the posts contained a subject actively exercising. A large number of posts focused on users’ bodies, including flat stomachs and muscles, often in a posed and sexualized manner. The implication of these findings is that a subset of fitspiration is focused on appearance. Previous research has noted that exercising for appearance-based purposes is associated with increased body image concerns and disordered eating symptomatology [31]. This content also suggests that being fit and healthy is equivalent to fitting in with current masculine and feminine body ideals; in many posts, fitness and beauty were depicted as being essentially the same concept.

More experimental and longitudinal research is needed to identify the impacts of fitspiration on both body image and exercise behavior, particularly for male users. However, in the context of the small body of experimental research [7] which indicates short-term harms of fitspiration in women, and qualitative research which indicates that some young female social media users have internalized messages about idealized bodies depicted in fitspiration [6], there may be a need to develop interventions to prevent such harms in the long term. It has been suggested that focusing on the benefits of fitness without also emphasizing thinness may offer promising results regarding body image and physical activity for young women [32]. Such an approach may also be worthwhile for young men, emphasizing the benefits of exercise without emphasizing muscularity.

The results of the content analysis in this study suggest that interventions to reduce potential harms of fitspiration could focus on critically analyzing objectifying messages in fitspiration, and other content aiming to inspire people to exercise and be healthy. Interventions aimed at women could aim to reduce pressures to be both thin and muscular and deconstruct the relationship between fitness, sexuality, and beauty; interventions aimed at men could focus on reducing the pressure to be muscular (particularly in the upper body). Further research could aim to develop these interventions for both general communities (eg, social media users) and clinical populations (eg, young people experiencing eating disorders). Furthermore, fitspiration communities appear to be most active on Instagram, suggesting that any social media–based interventions should focus primarily on Instagram, with a secondary emphasis on Tumblr and other platforms. However, due to the diverse nature of tagging on different social media sites, it is possible that fitspiration communities are very active on other social media platforms. Furthermore, fitspiration websites [20] should not be ignored when developing interventions.

Of note, coders also identified several themes that were not included in the analysis and have not been included in previous content analyses. These include: presence of professional fitness models; suspected instances of image manipulation; emphasis on back muscles; depiction of protein powder or supplements; tie-ins with particular products, companies, diets, or trends (eg, “clean eating,” “bikini bodies”); or depictions of particular subcommunities such as “fit mothers” and people tracking “fitness journeys.” Future research should consider these areas and the potential impact that they might have on male and female body image.

### Limitations

The authors acknowledge the limitations of this study. Although the study utilized 3 independent coders, the majority of variables included in the framework were subjective. Certain variables were nonspecific and only allowed simple yes or no coding. It is possible that our composite half hour did not accurately represent social media fitspiration; social media users may follow dedicated fitspiration blogs and pages rather than use hashtag-based searching, especially since searching for recent hashtags is difficult on some platforms. Fitspiration profiles may also be private and some posts would not have been extracted in our analysis. We only analyzed 1 hashtag (“#fitspo”) due to time resources; this hashtag was chosen over #fitspiration (which was used by Tiggemann and Zaccardo [21]) as #fitspo returned approximately 4 times the number of results. Although many posts analyzed also contained the #fitspiration hashtag, our analyses can only be interpreted as relating to this 1 hashtag. Little detail could be provided about videos as not all were able to be viewed; some of these videos were coded as single still images. Furthermore, in order to analyze similar styles of posts across platforms, only “most recent” posts were viewed. Although representative of posts uploaded and tagged within a particular time frame, we are unable to provide information about the “most popular” form of fitspiration.

### Conclusions

Overall, this study indicates that fitspiration on social media often encourages exercise in order to reach an appearance aligned with gendered body image ideals [8,13]. Our results also indicate that fitspiration imagery features men nearly as often as it features women and that men are just as likely to be objectified as women, albeit in different ways. These findings suggest a need to experimentally examine the impact of fitspiration-style posts on body image and exercise behaviors, including fitspiration and other fitness media aimed at men. If such research identifies harms of fitspiration, further research is also required to determine the best strategies to minimize potential harms. Interventions should focus on Instagram and Tumblr and consider their highly visual nature and mobile formats, and gendered body image messages.

## Appendix

Multimedia Appendix 1 Content Analysis Codebook.
