# Supplementary material for: “Fitspiration” on Social Media: A Content Analysis of Gendered Images
Source: J Med Internet Res. 2017 Mar 29;19(3):e95. doi: 10.2196/jmir.6368 (PMC5390113; doi:10.2196/jmir.6368)
Supplement: Multimedia Appendix 1 [file jmir_v19i3e95_app1.pdf]

Appendix 1 – Content analysis codebook

Categorical variables					
#	Variable name	κ	% agree	Description	Example
1	Relevancy	.73	93.7	Post is relevant to fitspiration (Y/N)	Content related to exercise, health, diet or body appearance
2	Platform	.98	99.5	Platform on which the post appears (Instagram, Twitter, Tumblr, or Facebook)	
3	Text	70.1	91.2	Post contains motivational or inspirational quote/text embedded in an image or as an image (Y/N)	'Unleash the beast' (grey text overlaying a muscular man lifting weights)
4	Caption	.75	92.5	Post contains a caption or other text added by the person who posted it (Y/N)	Text: 'Breakfast! Spinach, greek yoghurt, oats…' accompanying photo of breakfast
5	Photo	.8485	94.8	Post contains a photo (Y/N)	-
6	Video	.96	99.2	Post contains or links to a video (Y/N)	-
7	Selfie	.88	95.6	Post contains a selfie (Y/N)	-
8	Food	.91	97.2	Post's content thematically relates to food in some way (Y/N)	A photo of a meal, description of a meal plan
9	Exercise	.61	9383.7	Post's content thematically relates to exercise in some way (Y/N)	Text describing an exercise routine, photo in a gym
10	Face	.79	90.4	A person's face is visible in the post (Y/N)	-
11	Full body	.74	87.8	A person's whole body or nearly whole body is visible in the post (clothed or unclothed) (Y/N)	-
12	Stomach	.76	89.9	Post contains image emphasising subjects' stomach (Y/N)	-
13	Buttocks	.61	92.0	Post contains image emphasising subjects' buttocks (Y/N)	-
14	Sexualisation	.72	89.4	Post is sexual in nature (i.e. contains a 'sexual' or 'sexy' image, and/or references sex or being sexy) (Y/N)	Photo emphasising crotch/buttocks/cleavage, 'sexy' pose (e.g. posing in bikini)
15	Before/after	.66	99.0	Post references a person before and after weight loss/another body transformation (Y/N)	Photos of female subject 1 year apart, before and after getting visible abdominal muscles
16	Healthy eating	.70	89.9	Post emphasises eating 'healthy' food (Y/N)	Photo of beef curry with caption 'Healthy choices can still be made on vacation… #healthychoices'
17	Active exerciser	.80	88.1 90.4	Post contains image of a person before, during or after exercise (Y/N)	Selfie of sweaty man in gym changing room with caption 'Two and a half hour workout complete!'
Ordinal and interval variables					
#	Variable name	ρ		Description	Example
18	Thinness	.74	-	Thinness rating of main subject of image on Figure Rating Scale [20] (N/A, 1-9, Unable to assess)	-
1	Muscularity	.76	-	Muscularity rating of person	-
